# Estimation of Arsenic Content in Soil Based on Laboratory and Field Reflectance Spectroscopy

**DOI:** 10.3390/s19183904

**Published:** 2019-09-10

**Authors:** Lifei Wei, Ziran Yuan, Ming Yu, Can Huang, Liqin Cao

**Affiliations:** 1 Faculty of Resources and Environmental Science, Hubei University, Wuhan 430062, China; 2 Hubei Key Laboratory of Regional Development and Environmental Response, Hubei University, Wuhan 430062, China; 3 Faculty of Resources and Environmental Science, Wuhan University, Wuhan 430072, China

**Keywords:** soil arsenic content, hyperspectral remote sensing, characteristic bands, iteratively retaining informative variables, random forest regression, eXtreme gradient boosting regression

## Abstract

In this study, in order to solve the difficulty of the inversion of soil arsenic (As) content using laboratory and field reflectance spectroscopy, we examined the transferability of the prediction method. Sixty-three soil samples from the Daye city area of the Jianghan Plain region of China were taken and studied in this research. The characteristic wavelengths of soil As content were then extracted from the full bands based on iteratively retaining informative variables (IRIV) coupled with Spearman’s rank correlation analysis (SCA). Firstly, the IRIV algorithm was used to roughly select the original spectral data. Gaussian filtering (GF), first derivative (FD) filtering, and gaussian filtering again (GFA) pretreatments were then used to improve the correlation between the spectra and soil As content. A subset with absolute correlation values greater than 0.6 was then retained as the optimal subset after each pretreatment. Finally, partial least squares regression (PLSR), Bayesian ridge regression (BRR), ridge regression (RR), kernel ridge regression (KRR), support vector machine regression (SVMR), eXtreme gradient boosting (XGBoost) regression, and random forest regression (RFR) models were used to estimate the soil As values using the different characteristic variables. The results showed that, compared with the traditional method based on IRIV, using the characteristic bands selected by the IRIV-SCA method can effectively improve the prediction accuracy of the models. For the laboratory spectra experiment stage, the six most representative characteristic bands were selected. The performance of IRIV-SCA-SVMR was found to be the best, with the coefficient of determination (*R*^2^), root-mean-square error (RMSE), and mean absolute error (MAE) in the validation set being 0.97, 0.22, and 0.11, respectively. For the field spectra experiment stage, the 12 most representative characteristic bands were selected. The performance of IRIV-SCA-XGBoost was found to be the best, with the *R*^2^, RMSE, and MAE in the validation set being 0.83, 0.35, and 0.29, respectively. The accuracy and stability of the inversion of soil As content are significantly improved by the use of the proposed method, and the method could be used to provide accurate data for decision support for the treatment and recovery of As pollution over a large area.

## 1. Introduction

As a result of the increased use of heavy metals in industrial, agricultural, domestic, and technological applications, human exposure to heavy metals has risen dramatically. Heavy metals are difficult to degrade, easy to accumulate, and toxic. They can have an impact on crop growth, yield, and quality, and can be absorbed into food, thereby entering the food chain and posing a threat to human health [1]. The traditional approach to the monitoring of heavy metals in soil is laboratory monitoring, with the aid of atomic absorption spectroscopy [2], atomic fluorescence spectrometry [3], spectrophotometry [4], and other analytical methods based on optical instruments, which are time-consuming and costly on a large-area application. Although these methods have a high precision, the common conventional and laboratory-based techniques for determination are nonfield-portable, expensive, and need extra time for sample extraction and analysis [5]. The development of hyperspectral analysis technology has made it possible to use continuous, high-resolution spectral bands to predict the arsenic (As) content in soil, and efficient and rapid detection can be achieved.

Laser-induced breakdown spectroscopy (LIBS) is a fast and convenient method of field detection [6]. However, there are strict requirements for the collection, storage, transportation, and determination of samples, and any mistake in any one of the stages can cause errors in the measurement results. Secondary pollution of the samples can also easily occur [7]. Because hyperspectral data have a high resolution and multiple and continuous spectral bands, hyperspectral analysis technology can realize large-scale and rapid determination of soil heavy metal content, which saves human, material, and financial resources. This method avoids complicated sampling steps, and through the combination of sampling and comparison, it can greatly improve the reliability of the measurement of heavy metals in soil. At the same time, the development of remote sensing technology, especially hyperspectral remote sensing technology, provides us with a new way to realize large-scale monitoring [8,9]. Real-time on-line monitoring and the early warning of soil heavy metal pollution can effectively meet the requirements of precision agriculture. Therefore, it is of great practical significance to study the use of soil spectral analysis techniques, to quantitatively estimate the content of heavy metals in soil.

A number of researchers have achieved remarkable results using hyperspectral techniques to study soil heavy metals. Gholizadeh et al. [10] demonstrated that the support vector machine regression (SVMR) method for visible and near-infrared (Vis-NIR) spectra could be used directly for an accurate assessment of potentially toxic elements (PTEs), including copper (Cu), manganese (Mn), cadmium (Cd), zinc (Zn), iron (Fe), lead (Pb), and As concentrations. Moros et al. [11] combined NIR and attenuated total reflectance (ATR) mid-infrared (MIR) spectra with a multivariate partial least squares (MPLS) method, and simultaneously monitored 14 trace elements in the estuary of the Nerbioi-Ibaizabal River.

Zhang et al. [12] studied the statistical properties of different heavy metal elements and their correlation with different spectral transformation forms. The stepwise regression algorithm and the best fitness function F were used as indices to select the optimal bands, and the partial least squares regression (PLSR) method was used to construct the inversion model between the spectral reflectance in the different transformation forms and the heavy metal content. Zheng et al. [13] used the PLSR method to establish a model between the reflectance spectrum and the soil As content. Cross-validation was then used to prove the feasibility of using the reflectance spectra to invert the soil As content. Wang et al. [14] tested and analyzed the spectral curves in the range of 350 to 2500 nm, and established a multiple regression relationship model between the different soil spectral variables and the Cu content of the soil. Sun et al. [15] set the spectral bands associated with organic matter and clay minerals as the characteristic bands, with genetic algorithm based partial least squares regression (GA-PLSR) used to build the model, and the results of this study confirmed the huge potential of soil reflectance spectroscopy in estimating Zn concentration in soil. However, at present, the models used for regression in the literature are mostly linear models, and research combining machine learning models such as XGBoost is rare. Most of the studies of As content inversion are based on laboratory measurements, which cannot truly reflect the spectral response of soil in the field environment.

The purpose of this study was to explore the possibility of quantitatively detecting the As content in soil by laboratory and field reflectance spectroscopy, in order to find an efficient and convenient method. The specific objectives were to: (1) explore the characteristic bands of laboratory and field reflectance spectroscopy in As prediction; (2) model and analyze the different soil spectra by two characteristic band selection methods (iteratively retaining informative variables (IRIV) and IRIV coupled with Spearman’s rank correlation analysis (IRIV-SCA) and seven modeling methods; and (3) compare the performance of linear (Partial least squares regression (PLSR), bayesian ridge regression (BRR), ridge regression (RR)) and nonlinear ( kernel ridge regression (KRR), support vector machine regression (SVMR), eXtreme gradient boosting (XGBoost) regression, and random forest regression (RFR)) models for predicting As, attempting to achieve high feasibility and reliability.

## 2. Materials and Methods

### 2.1. Study Area

**Figure 1:**
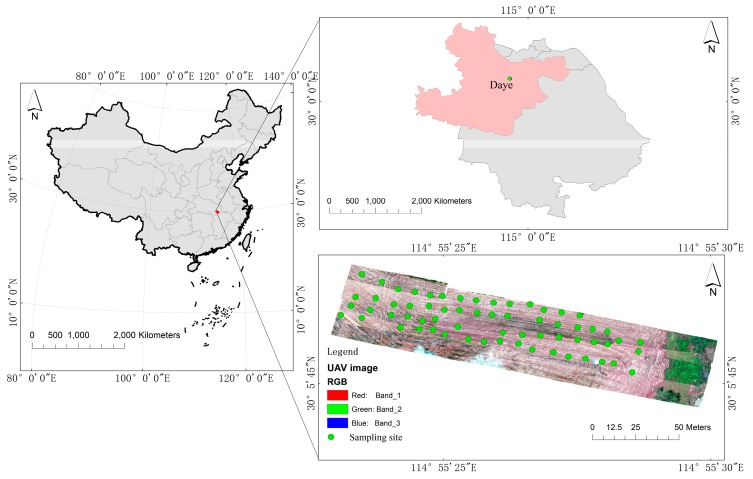
The location of the study area and the locations of the sampling (the unmanned aerial vehicle (UAV) image was taken by a DJI Matrice 600 Pro drone).

The city of Daye (114°31′−115°20′ E, 29°40′−30°15′ N) is located in the southeast of Hubei province, China, on the south bank of the middle reaches of the Yangtze River. Daye features a subtropical humid monsoon climate characterized by adequate sunlight, abundant rainfall, and mild temperature, the annual average temperature is 16.9 °C, and the average annual precipitation is 1385.8 mm. The city area is mainly hilly, with an altitude of 120–200 m. The administrative area covers 1566.3 km^2^. The Daye area is rich in mineral resources, and features a number of copper, iron, coal, and limestone mines [16], However, in recent years, the mining has greatly damaged the ecological environment, and the farmland soil (the main soil types are cinnamon soil and brown soil) near the mining area has been seriously polluted. Regarding field size (1 ha), the selected sample size had sufficient coverage of the predictor space and it was a suitable indicator of the population in which the models were applied [17]. The location of the study area and the locations of the sampling are shown in Figure 1.

### 2.2. Research Methods

#### 2.2.1. IRIV-SCA Characteristic Band Selection Algorithm

IRIV is a feature variable selection algorithm based on the binary matrix shuffling filter (BMSF) [18], in which a partial least squares model is established based on each row of the matrix, and the effects of different random variable combination models are evaluated using root-mean-squared error cross-validation (RMSECV) [19,20]. The IRIV algorithm requires multiple iterations. The purpose of each iteration is to retain the strongly informative variables and weakly informative variables, eliminate the uninformative variables and interfering variables, and finally identify the best variable set by backward elimination. The specific process is as follows:

*Step 1:* The raw data of m samples of p variables are formed into a matrix A containing only the numbers 0 and 1, where the number 1 represents a variable used for modeling, and the number 0 means that the variable was not used for the modeling. The RMSECV value obtained by five-fold cross-validation was used as the evaluation standard, and the vector of m×1 size was recorded as $RMSECV_{0}$. Substitute 1 in the *i*th column (*i* = 1, 2, …, p) of matrix A for 0, and 0 for 1 to obtain matrix B. The partial least squares (PLS) model is also established in each row of matrix B, and the vector of m×1 size is recorded as $RMSECV_{i}$.
*Step 2:* Define $\varphi_{0}$ and $\varphi_{i}$ to evaluate the importance of each variable as follows:
  (1) $$\varphi_{0k}=\{\begin{array}{c} k^{th}{RMSECV}_{0}A_{ki}=1\\ k^{th}{RMSECV}_{i}B_{ki}=1 \end{array};\varphi_{ik}=\{\begin{array}{c} k^{th}{RMSECV}_{0}A_{ki}=0\\ k^{th}{RMSECV}_{i}B_{ki}=0 \end{array}$$
  where $k^{th}$ represents the *k*th line in the vector, and the $k^{th}RMSECV_{0}$ and $k^{th}RMSECV_{i}$ represent the values of the *k*th row in the vectors $RMSECV_{0}$ and $RMSECV_{i}$, respectively. The mean values of $\varphi_{0}$ and $\varphi_{i}$ are denoted as $M_{i,in}$ and $M_{i,out}$, respectively, and the two mean values are subtracted to obtain $DMEAN_{i}$. If $DMEAN_{i}<0$, it is a strongly informative variable or a weakly informative variable; if $DMEAN_{i}>0$, it is an uninformative variable or an interfering variable.
  (2) $$DMEAN_{i}=M_{i,in}-M_{i,out}$$
  *P* = 0.05 was defined as the threshold for the Mann–Whitney U test [19,21], where the p value, denoted as *p_i_*, is computed by the Mann–Whitney U test with the distribution of $\varphi_{0}$ and $\varphi_{i}$. The smaller the $p_{i}$ value, the more significant the difference between the two distributions. Finally, the variables were divided into the four categories (strongly informative variables, weakly informative variables, uninformative variables, and interfering variables).
*Step 3:* Strongly informative variables and weakly informative variables are retained for each iteration, and uninformative variables and interfering variables are eliminated, so that a new subset of variables is generated. Return to step 1 for the next iteration until there are only strong and weak informative variables left. The defined variable types are listed in Table 1.
*Step 4:* The backward elimination of the reserved variables is undertaken as follows:
  (a) Denote t as the number of reserved variables.
  (b) For all the reserved variables, obtain the RMSECV value with five-fold cross-validation using PLS, which is denoted as $\theta^{t}$.
  (c) Leave out the *i*th variable and apply five-fold cross-validation to the remaining t−1 variables to obtain the RMSECV valu $\theta_{-i}$. Conduct this for all variables i=1,2,…,t.
  (d) If $\min\{\theta_{-i},1\leq i\leq t\}>\theta^{t}$, step (g) is performed.
  (e) When excluding the *i*th variable with the minimum RMSECV value, remove the *i*th variable and change t to be t−1.
  (f) Repeat steps (a) to (e).
  (g) The remaining variables are the final informative variables.
*Step 5:* The final informative variables are selected to form the matrix set $S=\left[x_{1},x_{2},\ldots ,x_{n}\right]$. $S=\left[x_{1},x_{2},\ldots ,x_{n}\right]$ are subject to Gaussian filtering (GF), first derivative (FD) filtering, and Gaussian filtering again (GFA), and the processed data and the soil samples are respectively subject to SCA. All the results are combined, and the top *k* numbers with the highest absolute values ($\left|r_{xy}\right|>0.6$) of correlation coefficients are selected. The corresponding data of GF, FD, and GFA are combined to obtain the *k* result sets with the best correlation as the characteristic bands.
  (a) The Gaussian filter (GF) [22] is a kind of linear smoothing filter which chooses weights according to the shape of a Gaussian function. It is very effective for suppressing noise obeying a normal distribution. The GF is expressed as shown in Equation (3):
    (3) $$g\left(\chi \right)=\frac{1}{\sqrt{2\pi \sigma }}\exp\left[-{\left(\frac{\chi }{2\sigma }\right)}^{2}\right]$$
    where *χ* is the distance of the weight function from the maximum point, and the scale parameter *σ* represents the width of the Gaussian function, which determines the smoothness of the filtering.
  (b) First derivative (FD) filtering can eliminate some baseline and other background noise, while improving the spectral resolution and sensitivity. It is widely used in spectral analysis [23].
    (4) $$S(\lambda_{i})=\frac{\left[\lambda_{i+1}-\lambda_{i}\right]}{2\Delta \lambda }$$
    where $\lambda_{i}$ represents the reflectance value of the *i*th band, Δλ represents the reflectance value of the next band, and Δλ is the wavelength interval.
  (c) Spearman’s rank correlation analysis (SCA) is used to describe the relationship between the soil spectral characteristics and the soil As content [24]. It evaluates the correlation of two statistical variables using a monotonic equation. SCA is expressed as shown in Equation (5):
    (5) $$r_{xy}=\frac{\sum_{i}^{N}\left(x_{i}-\bar{x}\right)(y_{i}-\bar{y})}{{\left[\sum_{i-1}^{N}{\left(x_{i}-\bar{x}\right)}^{2}\sum_{i-1}^{N}{\left(y_{i}-\bar{y}\right)}^{2}\right]}^{\frac{1}{2}}}$$
    where $x_{i}$ is the reflectance of the *i*th band, $y_{i}$ is the *i*th soil As content, $\bar{x}$ is the average of the band reflectance, and $\bar{y}$ is the average As content of the soil.
*Step 6:* StandardScaler [25] is used to calculate the mean and standard deviation of the training set so that the test data set can use the same transformation. The features are standardized by removing the mean and scaling to unit variance. Centering and scaling happen independently on each feature by computing the relevant statistics on the samples in the training set. The mean and standard deviation are then stored to be used on the test data using the transform method.
  (6) $$S=\frac{x-\mu }{\sigma }$$
  where x is the spectral matrix, μ is the standard deviation of the spectral matrix data, and σ is the mean of the spectral matrix data.

#### 2.2.2. Partial Least Squares Regression (PLSR)

PLSR is a new multivariate regression analysis method that can simultaneously achieve regression modeling [26], simplify the data structure, and analyze the correlation between two groups of variables, which brings great convenience to multivariate statistical analysis. The main difference with ordinary least squares regression is that PLSR adopts data dimension reduction, information synthesis, and screening techniques in the regression modeling process, and it can extract new integrated components that have the best explanatory power for the system, so that the model has better robustness.

#### 2.2.3. Bayesian Ridge Regression (BRR)

BRR assumes that the prior probability, the likelihood function, and the posterior probability are all normally distributed [27,28]. The prior probability is that the model output Y is a normal distribution with mean $X_{\theta }$, and the regularization parameter α is regarded as a random variable that needs to be estimated from the data. The prior distribution law of the regression coefficient θ is a spherical normal distribution with a hyperparameter λ. BRR estimates the hyperparameters α and λ and the regression coefficient θ by maximizing the marginal likelihood function.

#### 2.2.4. Ridge Regression (RR)

RR involves correcting the calculation formula of the estimated regression coefficients based on the “least squares principle” when constructing multiple linear regression models [29]. By abandoning the unbiased characteristic of the least squares method, it is more realistic and reliable to obtain regression coefficients at the cost of losing part of the information and reducing the accuracy.

#### 2.2.5. Kernel Ridge Regression (KRR)

KRR is a nonlinear regression method [30,31]. Using the nonlinear mapping function, the sample is mapped to the high-dimensional feature space, and the kernel function of the original space is used to replace the dot product operation of the high-dimensional feature space. The linear ridge regression is then conducted in the high-dimensional feature space.

#### 2.2.6. Support Vector Machine Regression (SVMR)

SVMR is the application of support vectors in the field of function regression [32,33]. There is only one class of sample point in SVMR, and the optimal hyperplane is not to divide the two types of sample points into the most open ones, but to minimize the total deviation of all the sample points from the hyperplane, when the sample points are between the two boundary lines.

#### 2.2.7. EXtreme Gradient Boosting Regression (XGBoost)

XGBoost is an optimized version of the gradient boosting algorithm. It can be applied to tasks such as classification, regression, sorting, etc. [34]. XGBoost uses the loss function to describe the second derivative of the function to be solved, adding a regular term to prevent overfitting. Attributes are sampled when building each tree, and the training speed is fast and the effect is good. The interior contains a large number of classification and regression trees, and the residuals are used to enhance the model.

#### 2.2.8. Random Forest Regression (RFR)

RFR is an integrated statistical learning classification and regression algorithm that combines multiple decision trees to produce similar predictions for different features for the same phenomenon [35,36]. The output is the average of all the decision tree results in a random forest. Assume that the training set is extracted independently from the distribution of the random vectors. The prediction result of the model is the mean of the *k* regression trees.

#### 2.2.9. Technical Process

**Figure 2:**
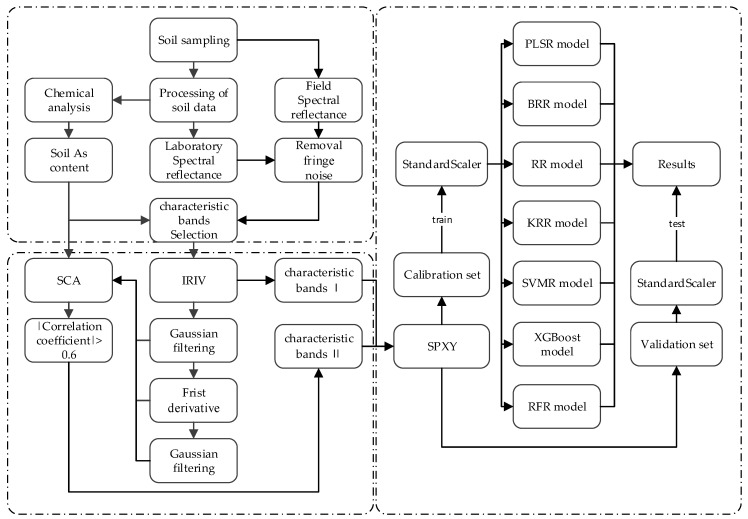
The technical flowchart of the algorithm proposed in this paper.

The IRIV method and the IRIV-SCA method were both used to select the characteristic bands, and the seven different regression methods were used to establish the As content prediction model. The specific algorithm flow is shown in Figure 2 and is summarized as follows. (1) The laboratory and field spectra were collected, respectively. (2) The characteristic bands were selected using the IRIV and IRIV-SCA methods. (3) Sample set partitioning based on joint *x*–*y* distances (SPXY) was used to partition the calibration set and validation set. Then, to avoid the importance of a feature being too large or too small, StandardScaler was used to standardize each column of the data. (4) The two sets of characteristic bands were modeled by the seven regression methods (PLSR, BRR, RR, KRR, SVMR, XGBoost, and RFR), and the best accuracy was obtained by comparative analysis.

### 2.3. Accuracy Evaluation

The prediction accuracy of the models was determined by the parameters of the coefficient of determination (*R*^2^), the root-mean-square error (RMSE), and the mean absolute error (MAE). *R*^2^ reflects the stability of the model establishment and verification. A larger *R*^2^ and a smaller RMSE and MAE indicates that the accuracy of the modeling, verification, and estimation, respectively, is higher. If *R*^2^ > 0.9, the prediction is excellent; if 0.82 ≤ *R*^2^ ≤ 0.9, the effect is good, and the established model can be used for actual detection; if 0.66 ≤ *R*^2^ < 0.82, the model can be used for approximate quantitative prediction; if 0.5 ≤ *R*^2^ < 0.66, the model is feasible to use but the prediction accuracy needs to be further improved; if *R*^2^ < 0.5, it is difficult to perform quantitative analysis of this component [37,38].

(7) $$R^{2}=1-\frac{\sum_{i-1}^{n}{\left(\hat{y_{i}}-y_{i}\right)}^{2}}{\sum_{i-1}^{n}{\left(y_{i}-\bar{y}\right)}^{2}}$$

(8) $$RMSE=\sqrt{\frac{\sum_{i-1}^{n}{\left(y_{i}-\hat{y_{i}}\right)}^{2}}{n}}$$

(9) $$MAE=\frac{1}{m}\sum_{i=1}^{n}\left|y_{i}-\hat{y_{i}}\right|$$

where *n* is the number of samples, $y_{i}$ is the measured value, $\hat{y_{i}}$ is the predicted value, and $\bar{y}$ is the average of the measured values.

### 2.4. Software

GF, FD, and SCA were programmed in MATLAB Version 2017b. SPXY, StandardScaler, and the regression models were written in Python/Jupyter Notebook. The machine learning algorithms in the scikit-learn packages were also used.

## 3. Experiments and Analysis

### 3.1. Experimental Procedure

#### 3.1.1. Soil Spectral Reflectance Measurement

In this paper, the soil of Daye, Hubei province, China, is taken as the research object. In this study area, field soil sampling, physicochemical analyses, and spectral collection and processing were conducted and two different methods of obtaining the soil spectra were used, one of which was laboratory based and the other was conducted in the field. In the field spectral measurement stage, an SVC HR-1024 field spectrometer was used to measure the spectra of the soil. The spectral resolution of this field spectrometer is as follows: 350 to 1000 nm is 1.5 nm, 1000 to 1900 nm is 3.8 nm, and 1900 to 2500 nm is 2.5 nm. The total number of bands is 990. Field spectral measurements were carried out on July 13, 2018, on a sunny day with a temperature of 36 °C, between 12:00 and 13:00 to ensure sufficient solar altitude angle, and the field of view angle of the probe was 25 degrees. Soils at relatively flat and open sites (avoiding plants, stones, etc.) were chosen as the target soils, and white-board calibration was performed on the spectrometer. Debris was removed from the soil surface before each measurement. The fiber optic probe was placed vertically at approximately 20 cm above the target and in the opposite direction to solar radiation. In order to eliminate the instability of the measurements, a 10 times average value was used as the average reflectivity of the soil sample. Three spectral curves (with each curve being the result of an average of 10 times) were saved for each soil sample, and the actual reflection data were obtained after arithmetic averaging.

In the laboratory spectral measurement stage, an ASD FieldSpec 3 field spectrometer was used to measure the spectra of the soil samples. The wavelength range of the ASD FieldSpec 3 field spectrometer is 350 nm to 2500 nm, with a spectral resolution of 1 nm. The total number of bands is 2151. The light source was a 1000-W halogen lamp with a 25-degree field of view angle, with the irradiation direction being 15 degrees from the vertical direction. The light source was set about 30 cm from the surface of the soil sample, with the probe perpendicular to the soil surface and about 10 cm away from the soil sample. White-board calibration was performed on the spectrometer before measurement. A 10 times average value was used as the average reflectivity of the soil sample, three spectral curves were saved for each soil sample, and the actual reflection data were obtained after arithmetic averaging.

#### 3.1.2. Soil Collection and Preparation

For the laboratory spectroscopy experiments, collection of the soil samples was necessary, but in the field experiments, this step was not needed. Sixty-three yellow-brown ploughed soil samples were collected by the method of chessboard-shaped sampling. The sampling depth was 0–15 cm. The foreign matter such as stones was removed during the collection, and the soil sample was collected by a four-point method, after being mixed well. Foreign bodies such as stones and plant roots in the dried soil were removed, and the soil was then crushed. The crushed soil was then passed through a 2-mm aperture sieve. The soil that passed through the 2-mm sieve was taken out by quartering and was roller-compacted to pass it through a 0.15-mm aperture sieve [39]. Each soil sample was then divided into two parts for spectral information collection and physical and chemical analysis.

#### 3.1.3. Chemical Analysis

A total of 63 samples were obtained for the physical and chemical analyses. The soil samples were digested with nitric acid/hydrochloric acid/perchloric acid and then measured with potassium borohydride/silver nitrate spectrophotometry. Each soil sample was measured three times, and the arithmetic mean was taken as the final As content in the soil.

### 3.2. Preprocessing of the Spectral Data

**Figure 3:**
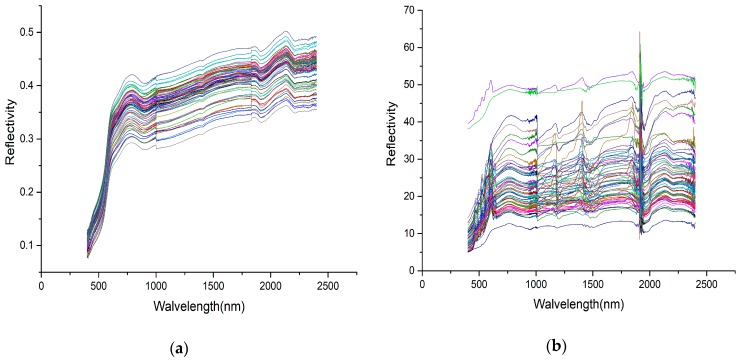
Soil reflectance spectra (with fringe noise removed) used to predict the As concentration in the soil: (**a**) laboratory reflectance spectra; (**b**) field reflectance spectra.

Due to the inevitable influence of factors such as the test environment, the instrument itself, the background of the sample, and stray light in the process of spectrum acquisition, wavelengths on the fringe of the Vis-NIR spectrometers contain relatively high noise. In order to reduce the external noise, the noisy edge bands of 350 to 399 nm and 2400 to 2500 nm were removed, and the 400 to 2399 nm wavelength was retained for the modeling analysis [40,41]. The soil reflectance spectra (with fringe noise removed) used to predict the As concentration in the soil are shown in Figure 3a,b.

### 3.3. Calibration Set and Validation Set

Before modeling, the samples needed to be grouped. One group was used for the establishment of the model, and is referred to as the calibration set, and the other group was used to test the predictability of the model, and is called the validation set. In this study, the gradient concentration method does not take into account the influence of spectral vectors, while the Kennard-Stone (KS) method does not take into account the concentration vectors [42]. In order to effectively cover the multidimensional vector space and improve the predictive ability of the established model, both the spectral vectors and concentration vectors were taken into account when partitioning the calibration set and validation set of samples. Therefore, the SPXY algorithm [43] was used to select 42 samples as the calibration set, and the remaining 21 samples were used as the verification set. As shown in Table 2, referring to the Soil Environmental Quality Risk Control Standard for Soil Contamination of Agricultural Land (GB15618-2018) in China, the average value for the Daye area is lower than the risk screening value for soil contamination of agricultural land, and so the Daye area belongs to the unpolluted area category.

## 4. Results

### 4.1. IRIV Characteristic Band Selection Algorithm

**Figure 4:**
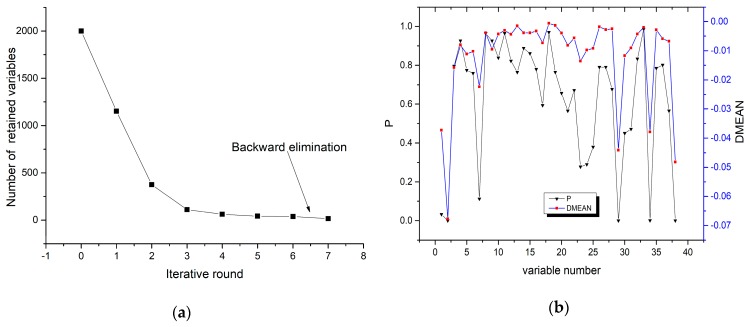
Iteratively retaining informative variables (IRIV) iterative process and wavelength type decision parameter values obtained using the laboratory spectral reflectance. (**a**) Number of retained variables in the iterative rounds of IRIV; (**b**) DMEAN and p value in the 6th iteration.

The purpose of the IRIV algorithm is to eliminate irrelevant variables and retain variables associated with soil As content. The algorithm uses the five-fold cross-validation method to establish the PLS model selection feature variables. The maximum principal factor in the PLS model is 10. In the laboratory spectra experiment stage, the IRIV algorithm was carried out for a total of seven rounds. As shown in Figure 4, the number of iterative variables in the first three rounds decreased rapidly, from 2000 variables to 489, and then the rate of variable reduction slowed down after this point. After the sixth iteration, the uninformative variables and interfering variables were completely eliminated. Generally speaking, only the strongly informative variables are selected as the optimal variable set. Although they have a significant positive effect, they are not always the optimal ones, because of the fact that the positive effect of the weakly informative variables is ignored. Thus, the weakly informative variables should be retained. The IRIV strategy is thus used to search for the significant variables through many rounds until no uninformative or interfering variables exist. The backward elimination operation was then carried out, and after the backward elimination of the seventh round, 15 characteristic bands related to soil As content were finally selected: 486 nm, 527 nm, 740 nm, 769 nm, 849 nm, 1033 nm, 1147 nm, 1184 nm, 1185 nm, 1241 nm, 1359 nm, 1365 nm, 2233 nm, 2336 nm, and 2382 nm. 

**Figure 5:**
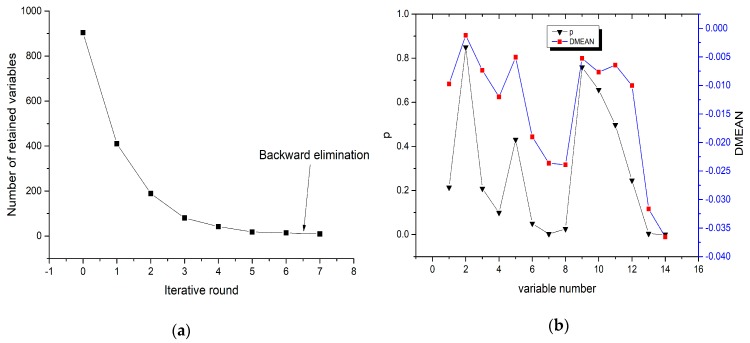
Iteratively retaining informative variables (IRIV) iterative process and wavelength type decision parameter values obtained using the field spectral reflectance. (**a**) Number of retained variables in the iterative rounds of IRIV; (**b**) DMEAN and p value in the 6th iteration.

In the field spectra experiment, the IRIV algorithm was performed for a total of seven rounds. As shown in Figure 5, the number of iterative variables in the first three rounds decreased rapidly, from 990 variables to 170. After six rounds of iteration, the uninformative variables and the interfering variables were completely eliminated, and the backward elimination operation was performed. After the backward elimination in the seventh round, nine characteristic bands related to soil As content were finally selected: 619.6 nm, 621 nm, 1186.8 nm, 1422.1 nm, 1871.7 nm, 1896.8 nm, 1907.5 nm, 2348.2 nm, and 2383.4 nm.

### 4.2. IRIV-SCA Characteristic Band Selection Algorithm

**Figure 6:**
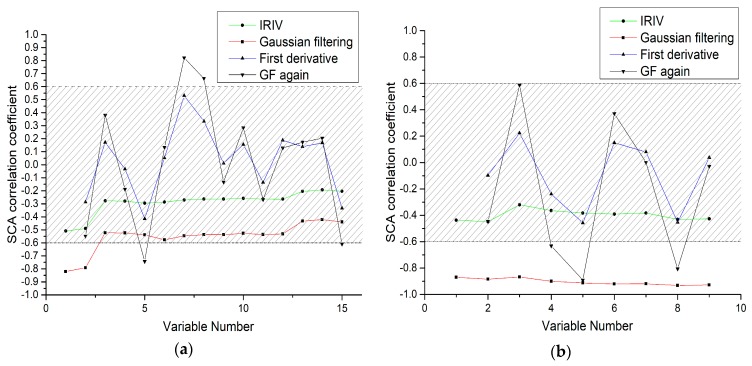
Correlation coefficients between the different pretreatments and the As concentration of soil. The green line indicates the IRIV spectral reflectance and the As concentration of soil, the red line indicates the Gaussian filtering (GF) spectral reflectance and the As concentration of soil, the blue line indicates the first derivative (FD) spectral reflectance and the As concentration of soil, and the black line indicates the GFA spectral reflectance and the As concentration of soil (**a**) Laboratory spectra of the soil samples; (**b**) Field spectra of the soil samples.

Considering that IRIV selects more characteristic variables, and IRIV also fails to change the original data, there may be cases where the unrelated variables are not completely eliminated and the original correlation is low. IRIV-SCA can not only eliminate all the irrelevant variables of the original full spectrum, but can also greatly reduce the number of independent variables, which can achieve the dual purpose of improving the accuracy of the algorithm and the efficiency of the execution. The GF, FD, and GFA preprocessing can effectively improve the correlation, and then SCA is used to find the correlation between the spectral data of each preprocessing and the As content of the soil. It can be seen from Figure 6 that the correlation coefficients of the original bands of the IRIV screening are generally below 0.6, but after the GF, FD, and GFA preprocessing, the correlation coefficients are improved to different degrees. The bands with an absolute value of correlation coefficient of greater than 0.6 after each pretreatment were extracted as the characteristic bands. The correlation of each feature band is shown in Table 3. For the laboratory spectra, a total of six characteristic bands (all the variables outside of the gray area) were selected. For the field spectra, a total of 12 characteristic bands were selected.

### 4.3. Analysis of the Results of the IRIV Feature Selection Algorithm

The characteristic bands obtained by the IRIV feature selection method were used for the modeling, and the data from the calibration set were used to build the model. To evaluate the prediction, the three parameters of coefficient of determination for prediction ($R_{p}^{2}$), root-mean-square error of prediction ($RMSE_{p}$), and mean absolute error of prediction ($MAE_{p}$) can be obtained by using the data of the verification set for prediction. The closer $R_{p}^{2}$ is to 1, the better the fit of the model and the better the stability of the model; the closer the values of $RMSE_{p}$ and $MAE_{p}$ are to 0, the higher the accuracy of the model and the better the predictive ability of the model [44]. Table 4 compares the accuracy of the seven different regression models in the laboratory and field conditions, respectively. The accuracy of the regression models based on the laboratory spectra is generally higher than that based on the field spectra. This is because the spectrometer is affected by the light source, water content, particle size, and other debris in the process of collecting data in the field, resulting in a messy spectral curve. For the laboratory spectra, BRR shows the highest accuracy; for the field spectra, RFR shows the highest accuracy. Overall, the accuracy of the models constructed using the characteristic bands selected by the original IRIV algorithm are generally low and cannot meet the actual needs.

### 4.4. Analysis of the Results of the IRIV-SCA Feature Selection Algorithm

It can be seen from Table 5 that the regression model constructed using IRIV-SCA to select the characteristic bands achieves a high inversion accuracy. For the laboratory spectra, SVMR obtains the highest prediction accuracy, with the $R_{p}^{2}$, $RMSE_{p}$, and $MAE_{p}$ of the validation set being 0.97, 0.22, and 0.11, respectively. For the field spectra, XGBoost obtains the highest prediction accuracy, with the $R_{p}^{2}$, $RMSE_{p}$, and $MAE_{p}$ of the validation set being 0.83, 0.35, and 0.29, respectively. This confirms that the IRIV-SCA feature selection algorithm can not only effectively improve the correlation between the spectral reflectance and soil As content, but it also greatly improves the inversion accuracy.

### 4.5. Model Performance

**Figure 7:**
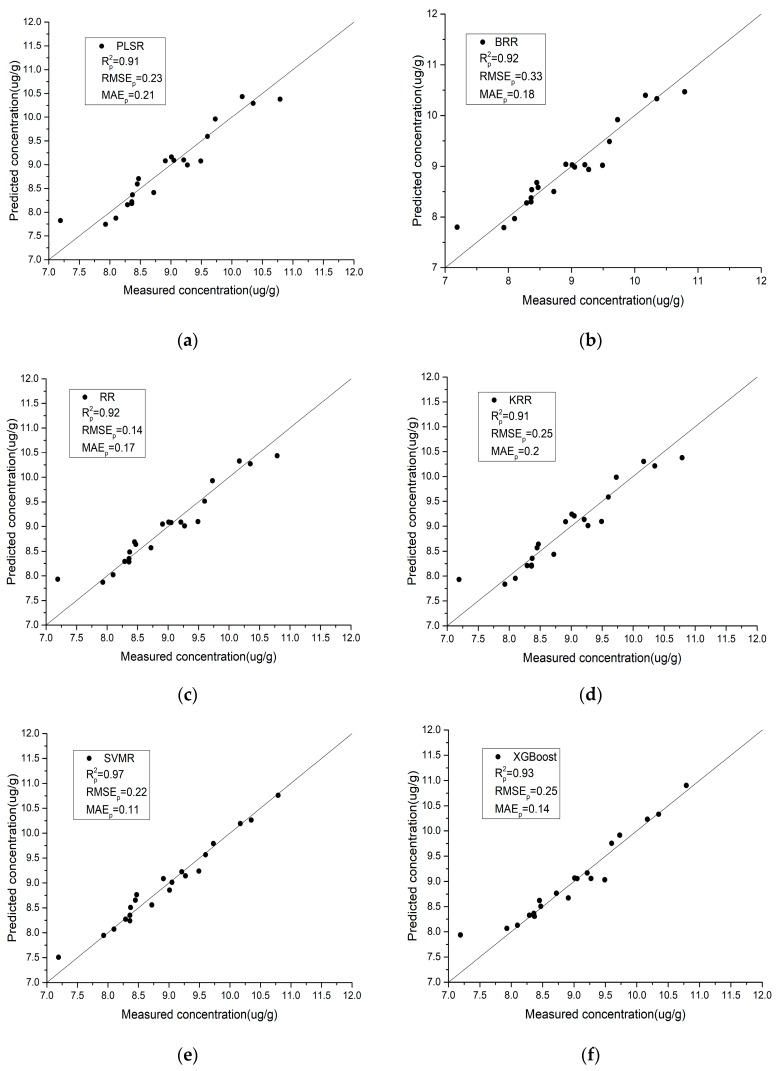

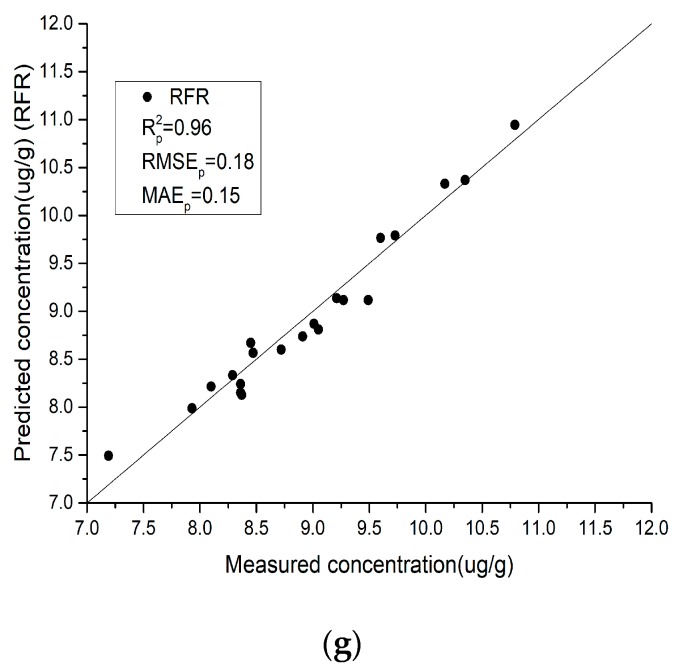
A comparison between the measured values and predicted values of the different regression models using laboratory spectra. (**a**) Partial least squares regression (PLSR); (**b**) Bayesian ridge regression (BRR); (**c**) ridge regression (RR); (**d**) kernel ridge regression (KRR); (**e**) support vector machine regression (SVMR); (**f**) eXtreme gradient boosting (XGBoost) regression; (**g**) random forest regression (RFR).

**Figure 8:**
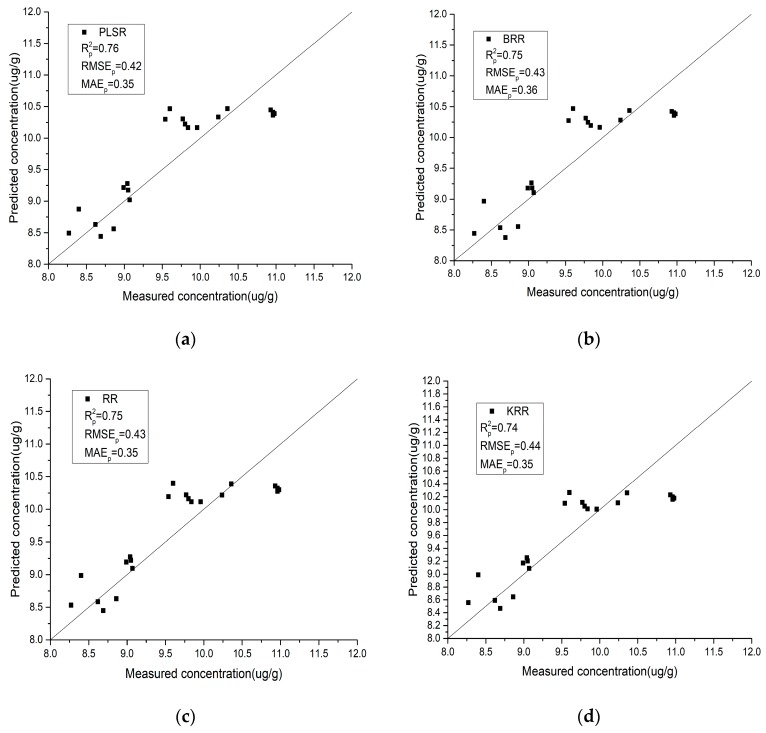

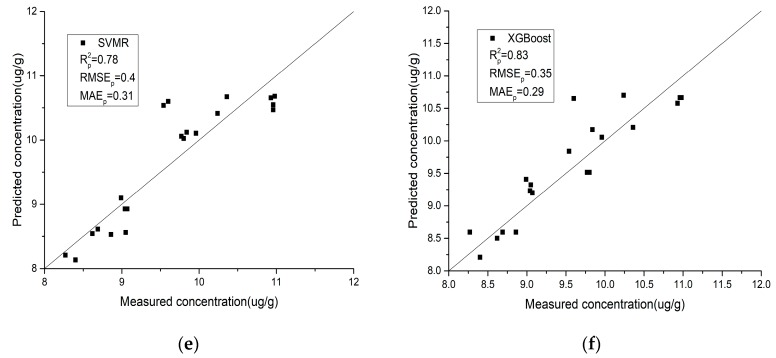

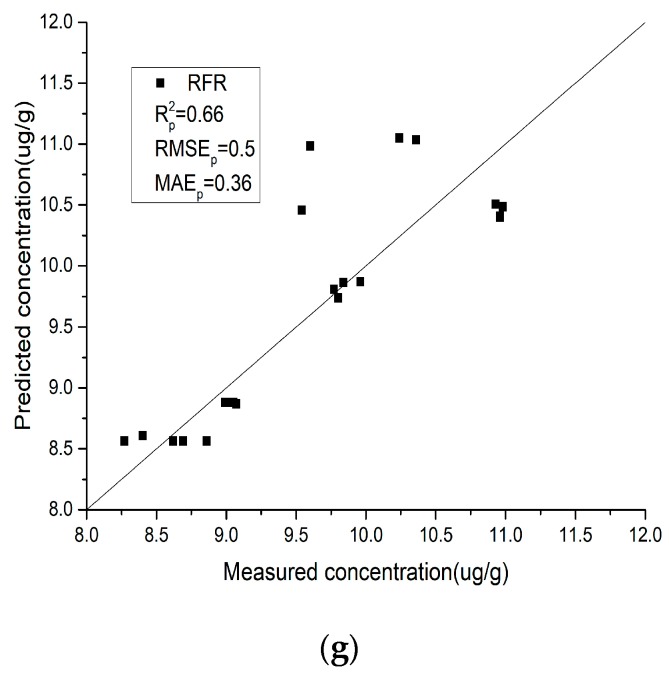
A comparison between the measured values and predicted values of the different regression models using field spectra. (**a**) Partial least squares regression (PLSR); (**b**) Bayesian ridge regression (BRR); (**c**) ridge regression (RR); (**d**) kernel ridge regression (KRR); (**e**) support vector machine regression (SVMR); (**f**) eXtreme gradient boosting (XGBoost) regression; (**g**) random forest regression (RFR).

Using the characteristic bands selected by IRIV-SCA, the relationship between the estimated and predicted values of the model validation set samples is shown in Figure 7 and Figure 8. The closer the scatter plots of the predicted and measured values are, the higher the accuracy of the model. By comparing the scatter plots of the different regression methods in the modeling process, the following conclusions can be drawn:

(1) Compared with the field spectra, the laboratory spectra are generally closer to the *y* = *x* line, which indicates that the laboratory spectra have better stability and predictive ability for the As content in soil. IRIV-SCA was used to intelligently select the characteristic bands, and the modeling accuracy and prediction accuracy of the model are both relatively high.
(2) For the laboratory spectra, SVMR obtains the highest *R*^2^ and the lowest RMSE and MAE values. This is shown in Figure 7e, where the black scatter points are located closest to the *y* = *x* line, and the trend is the most consistent with the *y* = *x* line. PLSR obtains the lowest *R*^2^ and the highest RMSE and MAE values. This is shown in Figure 7a, where the black scatter points are located close to the *y* = *x* line, but a few points exhibit slight deviations. For the field spectra, XGBoost obtains the highest *R*^2^ and the lowest RMSE and MAE values. This is shown in Figure 8f, where the black scatter points are located close to the *y* = *x* line and the trend is more consistent with the *y* = *x* line. RFR obtains the lowest *R*^2^ and the highest RMSE and MAE values. This is shown in Figure 8g, where the black scatter points exhibit large differences.

## 5. Discussion

Because the energy level transitions of the different functional group components in soil are different, the soil spectral curves also have different absorption and reflection characteristics. Therefore, it is possible to quantitatively analyze the soil As content by using spectral techniques. However, the soil spectra in the field environment are complex, and the soil parent material and external environmental influence parameters (soil moisture content, soil surface roughness, particle size factor, temperature factor, etc.) all have an effect on the spectral reflectance of the soil [45,46]. Lamine et al. [47] studied the potential effects of combining field and laboratory spectra with the data of Pb, Zn, Cu, and Cd in soil on the quantification and simulation of heavy metal soil pollution in floodplains. The results further demonstrated the feasibility of combining geochemistry analyses with field spectroradiometric data to generate models that can predict heavy metal concentrations. This finding is consistent with the conclusions of our study.

On the basis of the different measured spectra, a variety of soil As hyperspectral prediction models were established using different modeling methods. Compared with PLSR, both XGBoost and SVMR showed good modeling accuracy. This is mainly because PLSR is a linear method, and it does not perform well in solving nonlinear problems, while XGBoost and SVMR can better solve the problem of complex nonlinear relationships between independent variables and dependent variables.

When estimating soil As content using the original spectral reflectance obtained in the field, the results often vary greatly, due to factors such as soil moisture content, soil surface roughness, particle size, temperature, etc. After eliminating the influence of sample inconsistency by pre-treating the soil sample by air drying and sieving, the accuracy of estimating soil As content using laboratory spectral characteristics can be slightly improved. However, it is still impossible to accurately estimate soil As content. A large number of studies have proved that the correlation between spectral characteristics and soil properties can be significantly improved by transforming the original spectral values using FD and GF [48,49,50]. A combined preprocessing algorithm was used in this study. After the GF, FD, and GFA preprocessings, the correlation coefficients were improved, to different degrees. From the results, it can be concluded that this is a practical way to improve the estimation accuracy of soil As content. At the same time, more spectral transformations could be attempted in future research to find a better inversion index of soil As content.

The rapid and non-destructive estimation of the As content in soil is of great significance for soil pollution monitoring and precision agriculture. However, in hyperspectral full-band data, the band information is often redundant [51]. Spectral variable screening is a key step in soil hyperspectral research, which not only simplifies the model structure, but also eliminates irrelevant and low-contribution wavelength variables [52,53]. Although the traditional IRIV model can select the characteristic bands, in the face of the more complex environment in the field, it is affected by natural factors, and the problem of poor correlation of the original bands is apparent. Compared with the results of IRIV, both the laboratory and field accuracy are greatly improved. It is speculated that there are two reasons for this phenomenon: (1) A large number of studies have shown that different forms of spectral reflectance transformation can help to eliminate background interference and improve spectral sensitivity and correlation. In the process of spectral transformation of GF, FD, and GFA, some hidden spectral information in the original spectrum is exposed. Thus, the correlation between the spectrum and As content can be improved [49,50,54]. (2) SCA is a common way to extract sensitive bands, and the use of the higher correlation bands can significantly improve the stability and predictive ability of the model [53,55]. The IRIV-SCA feature selection algorithm combines the advantages of these two factors, has a strong generalization ability, it is able to effectively remove the influence of these factors, and can achieve better inversion results.

## 6. Summary and Conclusions

In this study, based on the spectral analysis of soil samples in both the laboratory and the field using hyperspectral techniques, 63 soil samples were collected. Based on the two different methods of selecting characteristic bands (IRIV and IRIV-SCA), seven different modeling methods were used (PLSR, BRR, RR, KRR, SVMR, XGBoost, and RFR). As a result, the best method for the inversion of the soil As content in this area was established, which will be of great significance for the monitoring of soil As content in this study region. The main conclusions are as follows:

The spectral reflectance of soil was measured in both the laboratory and in the field. In the field experiment, the soil was not air-dried, sieved, ground, etc., which was closer to the real application environment. The accuracy of the field-based model was lower than that of the model based on laboratory-measured spectra. The reason for this is that the acquisition of the measured spectral data is affected by the natural environment; however, the model based on field-measured spectral data has good stability and actual predictive ability, and has strong practicability.

IRIV and IRIV-SCA were both used to screen the characteristic bands. It was found that IRIV-SCA can effectively improve the correlation between the bands and soil As content, and can greatly improve the modeling accuracy. For the laboratory spectra experiments, the best experimental accuracy was improved from IRIV-BRR to IRIV-SCA-SVMR. For the field spectroscopy experiments, the best experimental accuracy varied from IRIV-RFR to IRIV-SCA-XGBoost. These results confirmed that the characteristic bands can be better extracted by the use of IRIV-SCA. The characteristics of soil spectral reflectance are the integrated effects of various physical and chemical properties, such as soil organic matter, acidity–alkalinity, moisture, salinity, and oxides. The results of this study will provide a basis for the large-scale retrieval of As in the soil of the Daye region in the future, and the approach could also be extended to other regions.

## Figures and Tables

**Table 1:** Variable classification rules.

Wavelength Variable Type	Classification Rules
Strongly informative	$DMEAN_{i}<0$, $P_{i}<0.05$
Weakly informative	$DMEAN_{i}<0$, $P_{i}>0.05$
Uninformative	$DMEAN_{i}>0$, $P_{i}>0.05$
Interfering	$DMEAN_{i}>0$, $P_{i}<0.05$

**Table 2:** Statistics of As concentrations for the collected soil samples.

Study Area	Dataset	Sample Size	Minimum (ug/g)	Maximum (ug/g)	Mean (ug/g)	SD	CV (%)	Skewness	Kurtosis
Daye	Entire	63	7.04	12.84	9.28	1.11	11.97%	0.58	0.41

**Table 3:** The feature bands and the correlation coefficients.

Algorithm	Spectral Type	Spectral Set (nm)	Correlation Coefficients
IRIV	Laboratory spectra	486, 527, 740, 769,849, 1033, 1147, 1184, 1185, 1241, 1359, 1365, 2233, 2336, 2382	−0.509, −0.490, −0.278, −0.279, −0.296, −0.287, −0.271, −0.264, −0.264, −0.259, −0.264, −0.264, −0.205, −0.194, −0.204
	Field spectra	619.6, 621, 1186.8, 1422.1, 1871.7, 1896.8, 1907.5, 2348.2, 2383.4	−0.437, −0.448, −0.320, −0.364 −0.383, −0.391, −0.383, −0.431, −0.427
IRIV-SCA	Laboratory spectra	GF_486_, GF_527_, GFA_849–769_, GFA_1147–1033_, GFA_1184–1147_, GFA_2382–2336_	−0.821, −0.792, −0.743, 0.822, 0.663, −0.609
	Field spectra	GF_619.6_, GF_621_, GF_1186.8_, GF_1422.1_, GF_1871.7_, GF_1896.8_, GF_1907.5_, GF_2348.2_, GF_2383.4_, GFA_1871.7–1422.1_, GFA_1896.8–1871.7_, GFA_2348.2__–__1907.5_	−0.870, −0.885, −0.868, −0.901, −0.913, −0.921, −0.919, −0.931, −0.929, −0.632, −0.892, −0.806

^1^ GF = Gaussian filtering; GFA = Gaussian filtering again

**Table 4:** Prediction accuracies of the As concentration obtained using laboratory spectra and field spectra based on IRIV.

Algorithm	Spectral Type	Models	Calibration Set			Validation Set		
			$R_{c}^{2}$	$RMSE_{c}$	$MAE_{c}$	$R_{p}^{2}$	$RMSE_{p}$	$MAE_{p}$
IRIV	Laboratory spectra	PLSR	0.29	0.94	0.73	0.52	0.67	0.49
		**BRR**	**0.91**	**0.34**	**0.26**	**0.79**	**0.44**	**0.36**
		RR	0.49	0.80	0.62	0.49	0.69	0.56
		KRR	0.55	0.76	0.59	0.48	0.70	0.56
		SVMR	0.99	0.11	0.10	0.59	0.62	0.49
		XGBoost	0.87	0.40	0.31	0.57	0.63	0.49
		RFR	0.78	0.53	0.39	0.27	0.82	0.69
	Field spectra	PLSR	0.27	1.00	0.75	0.37	0.74	0.62
		BRR	0.16	1.07	0.85	0.20	0.84	0.73
		RR	0.28	1.00	0.75	0.37	0.75	0.63
		KRR	0.29	0.99	0.75	0.42	0.72	0.60
		SVMR	0.75	0.59	0.32	0.23	0.83	0.64
		XGBoost	0.99	0.14	0.10	0.29	0.79	0.69
		**RFR**	**0.83**	**0.49**	**0.34**	**0.49**	**0.67**	**0.56**

**Table 5:** Prediction accuracies of the As concentration obtained using laboratory spectra and field spectra based on IRIV-SCA.

Algorithm	Spectral Type	Models	Calibration Set			Validation Set		
			$R_{c}^{2}$	$RMSE_{c}$	$MAE_{c}$	$R_{p}^{2}$	$RMSE_{p}$	$MAE_{p}$
IRIV-SCA	Laboratory spectra	PLSR	0.93	0.31	0.22	0.91	0.23	0.21
		BRR	0.94	0.30	0.19	0.92	0.33	0.18
		RR	0.93	0.31	0.19	0.92	0.14	0.17
		KRR	0.92	0.33	0.20	0.91	0.25	0.20
		**SVMR**	**0.98**	0.15	**0.11**	**0.97**	**0.22**	**0.11**
		XGBoost	0.98	0.13	0.01	0.93	0.25	0.14
		RFR	0.97	0.30	0.12	0.96	0.18	0.15
	Field spectra	PLSR	0.77	0.56	0.40	0.76	0.42	0.35
		BRR	0.78	0.55	0.38	0.75	0.43	0.36
		RR	0.77	0.56	0.37	0.75	0.43	0.35
		KRR	0.75	0.58	0.38	0.74	0.44	0.35
		SVMR	0.87	0.42	0.24	0.78	0.40	0.31
		**XGBoost**	**0.99**	**0.12**	**0.10**	**0.83**	**0.35**	**0.29**
		RFR	0.88	0.41	0.30	0.66	0.50	0.36
